# 

**Published:** 1890-07-12

**Authors:** 


SATURDAY, JULY 12th, 1890.

**The Princess and the First Thousand** - - - - 209

**Words of Consolation.**—The Burthen of Life - - - 210

**Right or Left ?** - - - - - - - - - 211

**The History and Capabilities of Herbal Simples.**—By W. T. Fernie, M.D. XIV.—Carraway—Carrot - 212

**Editor's Letter Box.**—North-Eastern Hospital for Children 212

**The Doctor's Pulpit.**—The Man and the Book - - - 213

**Working Women in Large Towns.**—VI.—Needlewomen—Fur Sewers, etc. - - - - - - - 214

**Everybody's Page** - - - - - - - - 215

**Notes and News** - - - - - - - - 216

**Annotations.**- International Hygiene—A Terrible Procession—State Logic - - - - - - - - 218

**The Practitioner's Mirror and Retrospect:**—

Medicine—Hot Air Treatment of Phthisis—Albuminaria - 219

Surgery—Arterial Suture—Cure of Arterio Venous Aneurism by Extirpation—Hepatic Surgery—Electric Prostration - - - - - - - - - 220

Therapeutics—Effects of Arsenic—A Substitute for Quinine—Podophyllin Poisoning—Powders in Papers - - - 221

New Drugs, Appliances, and Things Medical—Callard's Diabetic Foods, 221; Eucalyptus Oil in Infectious Diseases—On the Use of Anatto in Colouring Milk - - - - 222

**The New Lunacy Laws** - - - - - - - 223

**Passing Topics.**—A Fortunate Hospital - - - - 224

**Scraps and Gleanings—Books Received** - - - 224

### EXTRA SUPPLEMENT—THE NURSING MIRROR.

**En Passant.**—Bucks Nursing Home—Short Items—Pay or Be Paid?—Dundee Nursing Society—Ladies as Nurses—The Garden Party at Marlborough House - - - - - lvii

**The Fete of the First Thousand** - - - - - lviii

**Queen Victoria's Jubilee Institute for Nurses** - lxii

**Sketches of Insanity.**—By Francis H. Walmsley, M.D. II.—Causation of Insanity - - - - - - - lxiii

**A Day Off** - - - - - - - - - - lxiv

**The Nurse** (Not the Angel)- - - - - - - - lxv

**Beef Tea** - - - - - - - - - - lxv

**Everybody's Opinion.**—Words of Thanks—A Home of Rest lxvi

**Notes and Queries** - - - - - - - - lxvi

**Appointments—Vacancies** - - - - - - lxvi

**Amusements and Relaxation** - - - - - - lxvi

